# Frequency‐ and Layer‐Specific Modulation of Cortical Neuronal Activity by Pulsed Electrical Stimulation

**DOI:** 10.1002/mco2.70643

**Published:** 2026-02-19

**Authors:** Xinzhi Ye, Junfeng Wang, Jiao Liu, Zepeng Liu, Yuxin Huang, Wei Li, Jiaxin Wang, Xiyao Gu, Zhiyan Wang, Linlin Sun

**Affiliations:** ^1^ Department of Neurobiology School of Basic Medical Sciences Peking University Beijing China; ^2^ Key Laboratory for Neuroscience of Ministry of Education and National Health Commission of China Beijing China; ^3^ Department of Orthopedics Peking University International Hospital Beijing China; ^4^ Center of Medical and Health Analysis Peking University Health Science Center Beijing China; ^5^ Department of Global Health School of Public Health Peking University Beijing China; ^6^ School of Pharmaceutical Sciences (Shenzhen) Sun Yat‐sen University Shenzhen China; ^7^ Department of Anesthesiology School of Medicine Renji Hospital Shanghai Jiao Tong University Shanghai China; ^8^ Key Laboratory of Anesthesiology (Shanghai Jiao Tong University) Ministry of Education Shanghai China; ^9^ Institute of Brain Science and Brain‐inspired Research Shandong First Medical University & Shandong Academy of Medical Sciences Jinan China

**Keywords:** electrical stimulation, frequency‐specific modulation, neuronal synchrony, primary somatosensory cortex, two‐photon Ca^2+^ imaging

## Abstract

Electrical stimulation is a common technique in neuroscience and clinical therapies, with stimulation frequency being a critical factor in its efficacy. However, the cellular mechanisms by which different frequencies of pulsed electrical stimulation modulate neuronal activity remain poorly understood. In this study, we explore the effects of 60 Hz (low frequency [LF]) and 160 Hz (high frequency [HF]) pulsed electrical stimulation on excitatory and inhibitory neurons in the primary somatosensory cortex (S1) of mice using two‐photon Ca^2+^ imaging. Our results show that HF stimulation significantly increased Ca^2+^ activity in excitatory neurons in layer 2/3, both during and after stimulation, while LF stimulation enhanced neuronal activity only post‐stimulation. In layer 5 excitatory neurons, HF stimulation increased neuronal activity only after stimulation cessation, whereas LF stimulation transiently suppressed activity during stimulation. Both LF and HF stimulation enhanced activity in inhibitory neurons in layer 2/3 during stimulation. In summary, our study reveals that electrical stimulation activates both excitatory and inhibitory neurons, with its primary mechanism of action being the modulation of neuronal rhythm rather than the amplitude of their activity. These findings shed light on stimulation mechanisms, supporting its therapeutic potential for neuropsychiatric disorders targeting neuronal rhythmicity.

## Graphical abstract

High‐frequency electrical stimulation induces stronger activation in excitatory neurons, while both frequencies enhanced similar activation in inhibitory neurons. Both frequencies modulate neuronal rhythm and coherence in excitatory and inhibitory neurons similarly. These findings elucidate frequency‐specific modulation of excitatory and inhibitory neurons, providing a mechanistic foundation for optimizing therapeutic brain stimulation protocols.

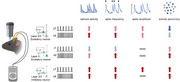

## 1 Introduction

Invasive and non‐invasive brain stimulation techniques, including deep brain stimulation (DBS), cortical stimulation, and transcranial electrical stimulation (TES), have been extensively studied for their ability to modulate neural activity in target brain regions for both neuroscience research and clinical applications. These techniques involve the application of electrical pulses to influence brain circuits and are increasingly used to investigate brain function as well as to treat neurological and psychiatric disorders [1, 2, 3, 4, 5, 6, 7, 8]. Common stimulus waveforms in these methods are pulsed, and it has been shown that pulsed electrical stimulation can effectively regulate neural oscillations and related neural circuits [9, 10]. Frequency, in particular, is a critical parameter that determines the efficacy of electrical stimulation in therapeutic contexts. For example, high‐frequency DBS (e.g., 130 Hz or 160 Hz) of the subthalamic nucleus (STN) is widely employed to treat tremors in patients with Parkinson's disease, while low frequency (e.g., 60 Hz) of the STN has been found to improve symptoms such as hypokinetic dysarthria and gait freezing [10, 11, 12]. These findings suggest that varying frequencies of pulsed electrical stimulation have distinct effects on both clinical symptoms and underlying neural mechanisms. However, despite its widespread use, the cellular‐level mechanisms by which different frequencies of electrical pulses modulate neuronal activity remain poorly understood.

Recent studies have shown that evoked neuronal responses by electrical stimulations depend nonlinearly on stimulation frequency, and their dynamics vary with distance from the electrode, ranging from sustained activation nearby to transient activation at distal sites, often followed by post‐stimulation suppression [13]. Further studies revealed that continuous electrical stimulation recruits neurons with distinct temporal profiles depending on frequency and location, with higher frequencies leading to more localized responses over time [14]. At the microscale, electrical stimulation induces dense Ca^2+^ and glutamate responses within 20–40 µm of the electrode, but sustained glutamate release can outlast stimulation [15]. Moreover, although both excitatory and inhibitory neurons are recruited, they exhibit distinct spatial patterns. The evoked responses in excitatory neurons are more strongly modulated by pre‐stimulus activity, indicating that the initial brain state could also be a key variable [16].

Despite these advances, critical gaps remain: first, the cell‐type and layer‐specific mechanisms of frequency effects are unclear, leaving a key question unanswered: Why is HF stimulation clinically more effective for certain symptoms? Second, it is unknown whether electrical stimulation modulates neural activity primarily through rhythmic entrainment or mere changes in intensity. This question is particularly relevant given that pathological conditions are often characterized by aberrant neuronal synchrony; for example, hypersynchronization in superficial cortical layers is a hallmark of chronic pain [17, 18], while excessive beta‐band synchronization is a core pathophysiology of Parkinson's disease [19]. Lastly, the impact of electrical stimulation on neuronal synchrony across layers and cell types has not been established.

Herein, we employed two‐photon Ca^2+^ imaging to investigate 60 Hz low‐frequency (LF) and 160 Hz high‐frequency (HF) pulsed electrical stimulation in mouse primary somatosensory cortex (S1). Our results revealed frequency‐ and layer‐specific modulation: HF enhanced layer 2/3 excitatory neuron activity during and post‐stimulation, while LF exerted delayed enhancement; layer 5 excitatory neurons were more excitation resistant, with HF increasing activity only post‐stimulation and LF transiently suppressing it during stimulation; both frequencies activated layer 2/3 inhibitory neurons. Together, our study reports a frequency‐ and layer‐specific logic governing how pulsed electrical stimulation modulates cortical neurons, providing a new cellular‐level explanation for its efficacy and frequency‐dependent effects.

## 2 Results

### 2.1 HF Enhanced Excitatory Neuronal Activity in Layer 2/3 During Stimulation

**FIGURE 1:**
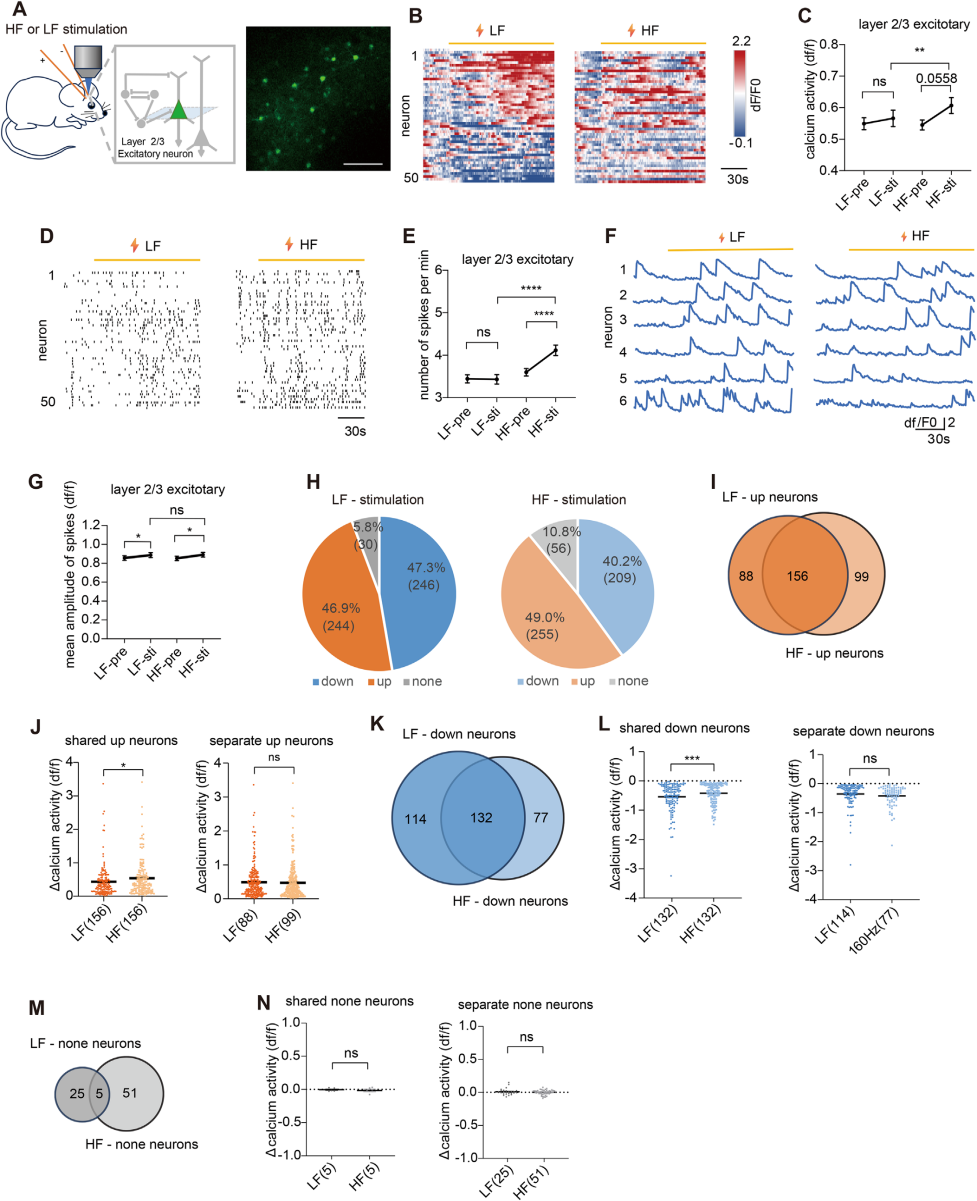
HF enhanced excitatory neuronal activity in layer 2/3 during stimulation. (A) Scheme of the experimental design and the example of two‐photon image of layer 2/3 excitatory neurons. Scale bar, 100 µm. (B) Example of heatmap of Ca^2+^ activity from neurons before and during electrical stimulation (*n* = 50). (C) Ca^2+^ activity of layer 2/3 excitatory neurons before and during stimulation (*n* = 520 neurons from four mice). (D) Ca^2+^ activity spikes from excitatory neurons in layer 2/3 before and during stimulation. (E) Numbers of spikes per minute of layer 2/3 excitatory neurons before and during stimulation (*n* = 520 neurons from four mice). (F) Example of Ca^2+^ traces from six neurons before and during stimulation. (G) Mean amplitude of spikes of layer 2/3 excitatory neurons before and during stimulation (*n* = 520 neurons from four mice). (H) Pie charts of the number and proportion of neurons whose Ca^2+^ activity increased, remained unchanged, and decreased when stimulated at LF or HF. (I) Venn diagram of layer 2/3 excitatory neurons with increased Ca^2+^ activity during LF or HF stimulation. (J) Δ Ca^2+^ activity of shared Up neurons and separate Up neurons during LF or HF stimulation. (K) Venn diagram of layer 2/3 excitatory neurons with decreased Ca^2+^ activity during LF or HF stimulation. (L) Δ Ca^2+^ activity of shared Down neurons and separate Down neurons during LF and HF stimulation. (M) Venn diagram of layer 2/3 excitatory neurons with no change during LF or HF stimulation. (N) ΔCa^2+^ activity of shared none neurons and separate none neurons during LF or HF stimulation. *p < 0.05, **p < 0.01, ***p < 0.001, ****p < 0.0001. Data are represented as mean ± SEM.

To examine the effect of electrical stimulation on excitatory and inhibitory neurons, we microinjected AAV‐CaMKIIα‐GCaMP6s and AAV‐GAD67‐GCaMP6s into the S1 and employed in vivo two‐photon Ca^2+^imaging to examine the Ca^2+^ activity of excitatory neurons in layer 2/3 and layer 5, and inhibitory neurons in layer 2/3 before, during, and after electrical stimulations. Stimulating electrodes were implanted within 50 µm of the imaging plane (Figure 1A). The Ca^2+^ activity was recorded over a course of 6‐min electrical stimulation (2‐min baseline, 2‐min stimulation on, and 2‐min stimulation off) at 60 Hz (LF) and 160 Hz (HF).

We first examined the effects of LF and HF stimulations on layer 2/3 pyramidal neurons during the stimulation delivery (stimulation on vs. baseline). HF stimulation induced a trend toward increased averaged Ca^2^^+^ activity (*p* = 0.056; Figure 1B,C), significantly increased the spike frequency (*p* < 0.001; Figure 1D,E), and the peak amplitude (*p* < 0.050; Figure 1F,G). In contrast, LF stimulation only increased the peak amplitude (*p* < 0.050; Figure 1F,G), but had no significant impact on the averaged Ca^2^^+^ activity and the spike frequency. To further analyze the differences between HF and LF stimulation, neurons with activity increases exceeding 10% above baseline were classified as “Up neurons,” and those with decreases exceeding 10% were classified as “Down neurons.” Both LF and HF stimulation increased activity in a similar number of neurons (Figure 1H), with the majority being the same neurons (156/244 for LF and 156/255 for HF; Figure 1I). However, HF stimulation elicited a greater activity increase in these 156 shared Up neurons (*p* = 0.013; Figure 1J), indicating that HF stimulation was more effective in enhancing neuronal activity. Similarly, LF and HF stimulation decreased activity in a comparable number of neurons (Figure 1H), with most being the same neurons (132/246 for LF and 132/209 for HF; Figure 1K). However, HF stimulation caused less activity reduction in these 132 shared Down neurons (*p* < 0.001; Figure 1L), suggesting that HF stimulation was less effective at suppressing activity. The remaining neurons showed no significant changes under either LF or HF stimulation (Figure 1M,N). In summary, LF stimulation increased and decreased activity in distinct neural populations, resulting in no net change in overall activity during stimulation. In contrast, HF stimulation produced greater activity increases and smaller decreases in a largely overlapping neural population compared to LF stimulation, leading to an overall elevation in activity during stimulation.

### 2.2 Both HF and LF Enhanced Excitatory Neuronal Activity in Layer 2/3 After Stimulation Cessation

**FIGURE 2:**
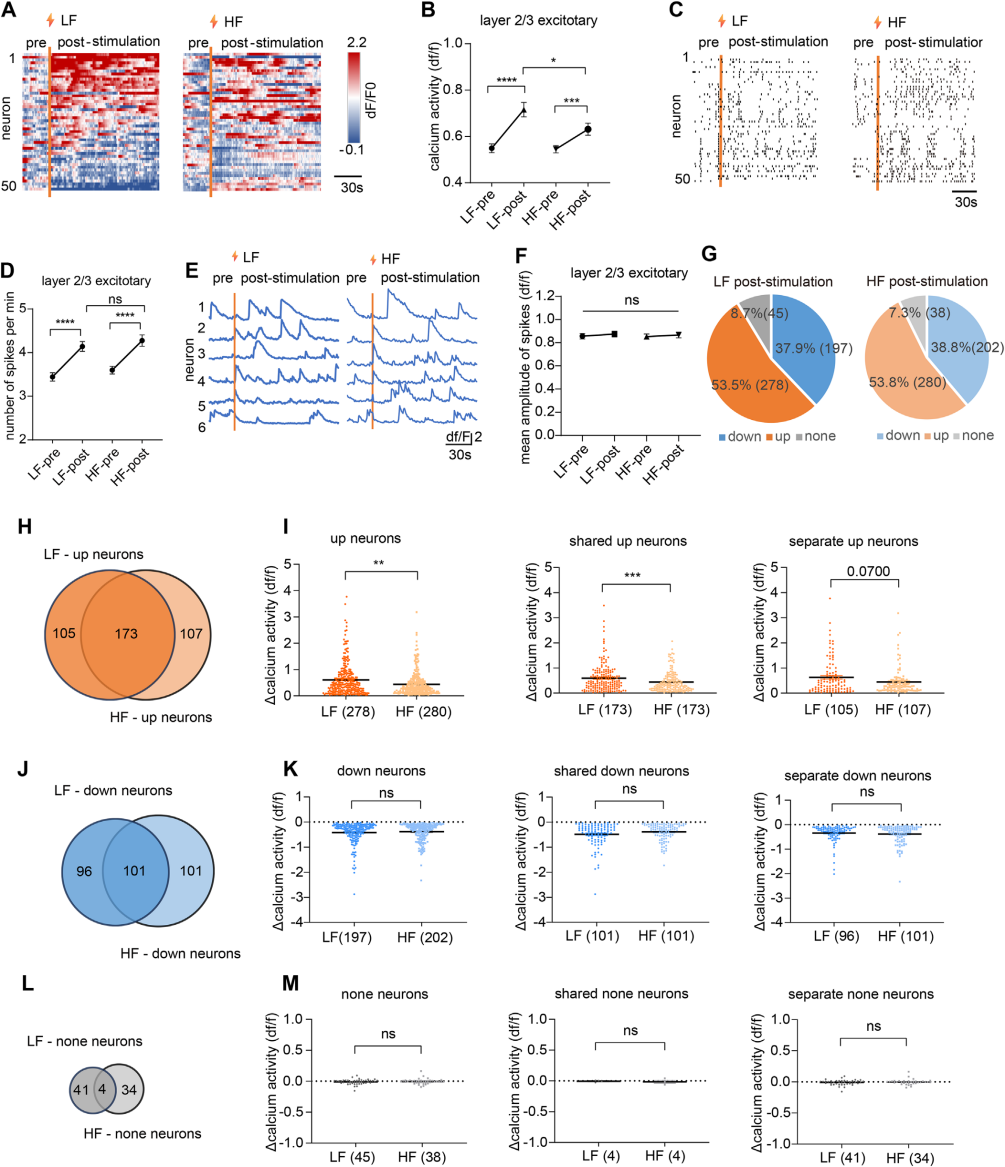
Both HF and LF enhanced excitatory neuronal activity in layer 2/3 after stimulation cessation. (A) Heatmap of Ca^2+^ activity from 50 neurons before and after stimulation. (B) Ca^2+^ activity of layer 2/3 excitatory neurons before and after stimulation (*n* = 520 neurons from four mice). (C) Ca^2+^ activity spikes from 50 excitatory neurons in layer 2/3 before and after stimulation. (D) Numbers of spikes per minute of layer 2/3 excitatory neurons before and after stimulation (*n* = 520 neurons from four mice). (E) Example of Ca^2+^ traces from six neurons before and after stimulation. (F) Mean amplitude of spikes of layer 2/3 excitatory neurons before and after stimulation (*n* = 520 neurons from four mice). (G) Pie charts of the number and proportion of neurons whose Ca^2+^ activity increased, remained unchanged, and decreased respectively after stimulation at LF or HF. (H) Venn diagram of layer 2/3 excitatory neurons with increased Ca^2+^ activity after LF or HF stimulation. (I) Δ Ca^2+^ activity of Up neurons, shared Up neurons, and separate Up neurons after LF or HF stimulation. (J) Venn diagram of layer 2/3 excitatory neurons with decreased Ca^2+^ activity after LF or HF stimulation. (K) ΔCa^2+^ activity of Down neurons, shared Down neurons, and separate Down neurons after LF or HF stimulation. (L) Venn diagram of layer 2/3 excitatory neurons staying unchanged after LF or HF stimulation. (M) Δ Ca^2+^ activity of unchanged neurons, shared unchanged neurons, and separate unchanged neurons after LF or HF stimulation. *p < 0.05, **p < 0.01, ***p < 0.001, ****p < 0.0001. Data are represented as mean ± SEM.

We then examined the effects of LF and HF stimulation on layer 2/3 pyramidal neurons after the termination of stimulation (stimulation off vs. baseline) to evaluate any lasting effect of a short electrical stimulation. Compared to pre‐stimulation, the averaged Ca^2+^ activities after the cessation of both LF and HF were higher (*p* < 0.001; Figure 2A,B), with activities during post‐LF even higher than those during post‐HF (*p* = 0.013; Figure 2A,B). Spike frequencies during both post‐LF and ‐HF were elevated (*p* < 0.001; Figure 2C,D), but peak amplitude remained unchanged (Figure 2E,F). Response patterns of these 520 neurons were also analyzed. Both LF and HF stimulation also increased activity in a similar number of neurons (Figure 2G), with the majority being the same neurons (173/278 for LF and 173/280 for HF; Figure 2H). The shared Up and separated Up neurons displayed higher Ca^2+^ activity during post‐LF compared to post‐HF (Up neurons, *p* = 0.001; Shared Up neurons, *p* < 0.001; Separate Up neurons, *p* = 0.070; Figure 2I), indicating that LF stimulation had a delayed effect in enhancing neuronal activity. Similarly, LF and HF stimulation decreased activity in a comparable number of neurons (Figure 2G), with most being the same neurons (101/197 for LF and 101/202 for HF; Figure 2J). These shared Down and separated Down neurons displayed comparable Ca^2+^ activity during post‐LF and post‐HF (Figure 2K). No significant changes in the remaining neurons were observed post either LF or HF stimulation (Figure 2L,M). In summary, for the S1 layer 2/3 excitatory neurons, both LF and HF continue to enhance the Ca^2+^ activity after stimulation cessation, with LF being more potent during this stage. Given that HF stimulation robustly increased neuronal activity both during and after stimulation, LF stimulation primarily enhanced activity post‐stimulation and enhanced the activity of approximately 50% neurons during stimulation (though no net rise in overall activity); these findings indicate that electrical stimulation could effectively boost excitatory neuron activity, with higher frequencies producing stronger and more immediate effects.

### 2.3 HF and LF Did Not Elevate Excitatory Neuronal Activity in Layer 5 During Stimulation

**FIGURE 3:**
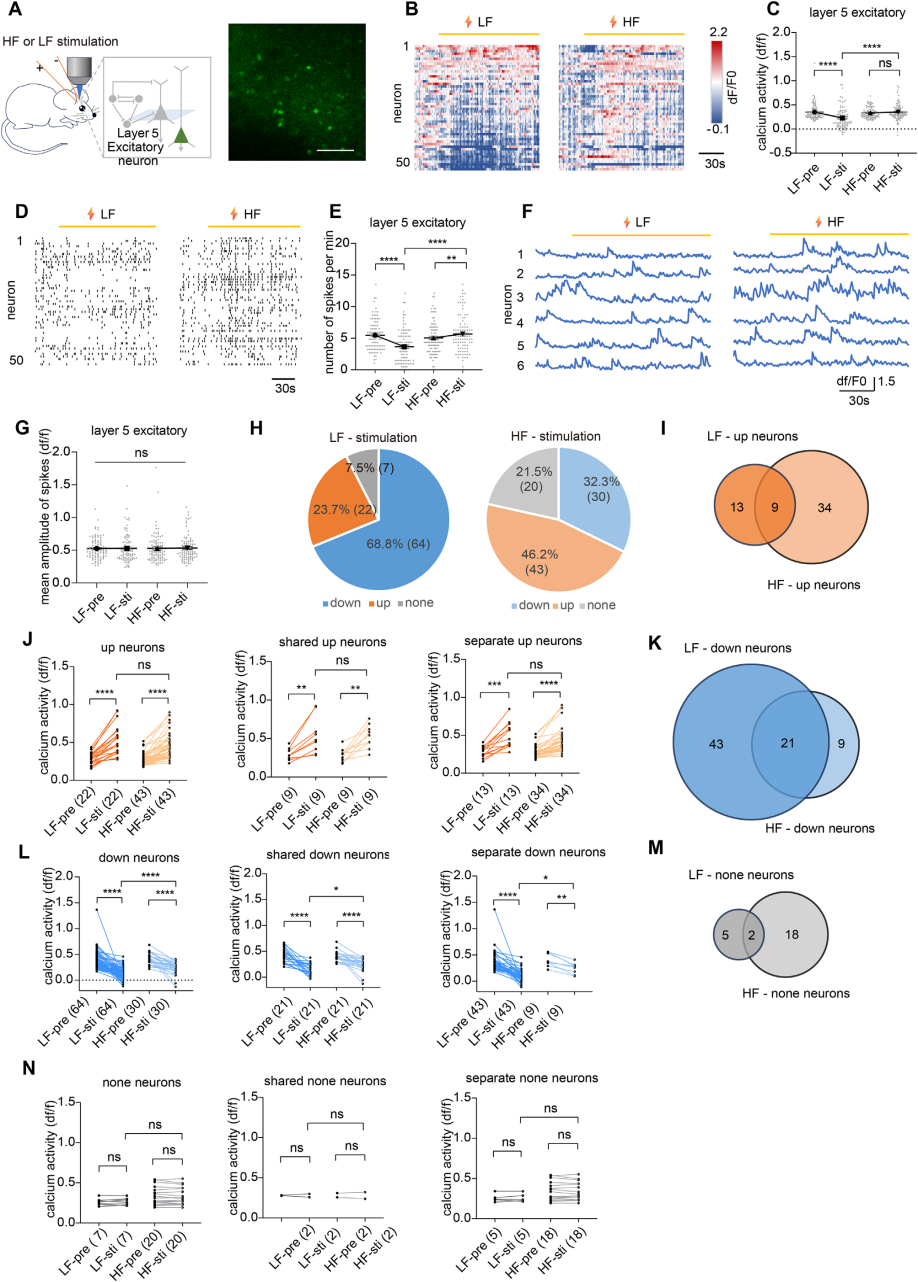
HF and LF did not elevate excitatory neuronal activity in layer 5 during stimulation. (A) Scheme of the experimental design and the example of two‐photon image of layer 5 excitatory neurons. Scale bar, 100 µm. (B) Example heatmap of Ca^2+^ activity from 50 neurons before and during pulse stimulation. (C) Ca^2+^ activity of layer 5 excitatory neurons before and during stimulation (*n* = 93 neurons from one mouse). (D) Ca^2+^ activity spikes from 50 excitatory neurons in layer 5 before and during stimulation. (E) Numbers of spikes per minute of layer 5 excitatory neurons before and during stimulation (*n* = 93 neurons from one mouse). (F) Example of Ca^2+^ traces from six neurons before and during stimulation. (G) Mean amplitude of spikes before and during stimulation (*n* = 93 neurons from one mouse). (H) Pie charts of the number and proportion of neurons whose Ca^2+^ activity increased, remained unchanged, and decreased during LF or HF stimulation. (I) Venn diagram of layer 5 excitatory neurons with increased Ca^2+^ activity during LF or HF stimulation. (J) Ca^2+^ activity of Up neurons, shared Up neurons, and separate Up neurons before and during LF or HF stimulation. (K) Venn diagram of layer 5 excitatory neurons with decreased Ca^2+^ activity during LF or HF stimulation. (L) Ca^2+^ activity of Down neurons, shared Down neurons, and separate Down neurons before and during LF or HF stimulation. (M) Venn diagram of layer 5 excitatory neurons with no change during LF or HF stimulation. (N) Ca^2+^ activity of none neurons, shared none neurons, and separate none neurons before and during LF or HF stimulation. *p < 0.05, **p < 0.01, ***p < 0.001, ****p < 0.0001. Data are represented as mean ± SEM.

We first examined the effects of electrical stimulations on layer 5 pyramidal neurons during the stimulation delivery. HF did not change the overall activity but increased the spike frequency during stimulation (*p* = 0.328; Figure 3B,C; *p* = 0.004, Figure 3D,E), and LF significantly reduced the overall Ca^2+^ activity (*p* < 0.001; Figure 3B,C) and spike frequency (*p* < 0.001; Figure 3D,E), but not peak amplitude (Figure 3F,G). Analysis of the neural response patterns revealed LF‐ and HF‐induced activity change in distinct populations, with Up neurons being the majority under HF but Down neurons being the majority under LF (Figure 3H). Shared Up and separated Up neurons displayed comparable Ca^2+^ activity increase during HF and LF stimulations (*p* = 0.882 for shared Up neurons, *p* = 0.069 for separate Up neurons; Figure 3I,J), while shared Down and separate Down neurons were more robustly inhibited under LF (*p* = 0.032 for shared Down neurons, *p* = 0.010 for separate Down neurons; Figure 3K,L). The remaining neurons showed no significant changes during both stimulations (Figure 3M,N). In summary, during the stimulation phase, HF produced no net increase in total activity of layer 5 excitatory neurons, while LF induced slight overall inhibition. Given that layer 2/3 neurons showed net increase in HF and no change in LF during stimulation, these results suggest that layer 5 pyramidal neurons have greater resistance to excitation compared to superficial layers.

### 2.4 HF Enhanced Excitatory Neuronal Activity in Layer 5 After Stimulation Cessation

**FIGURE 4:**
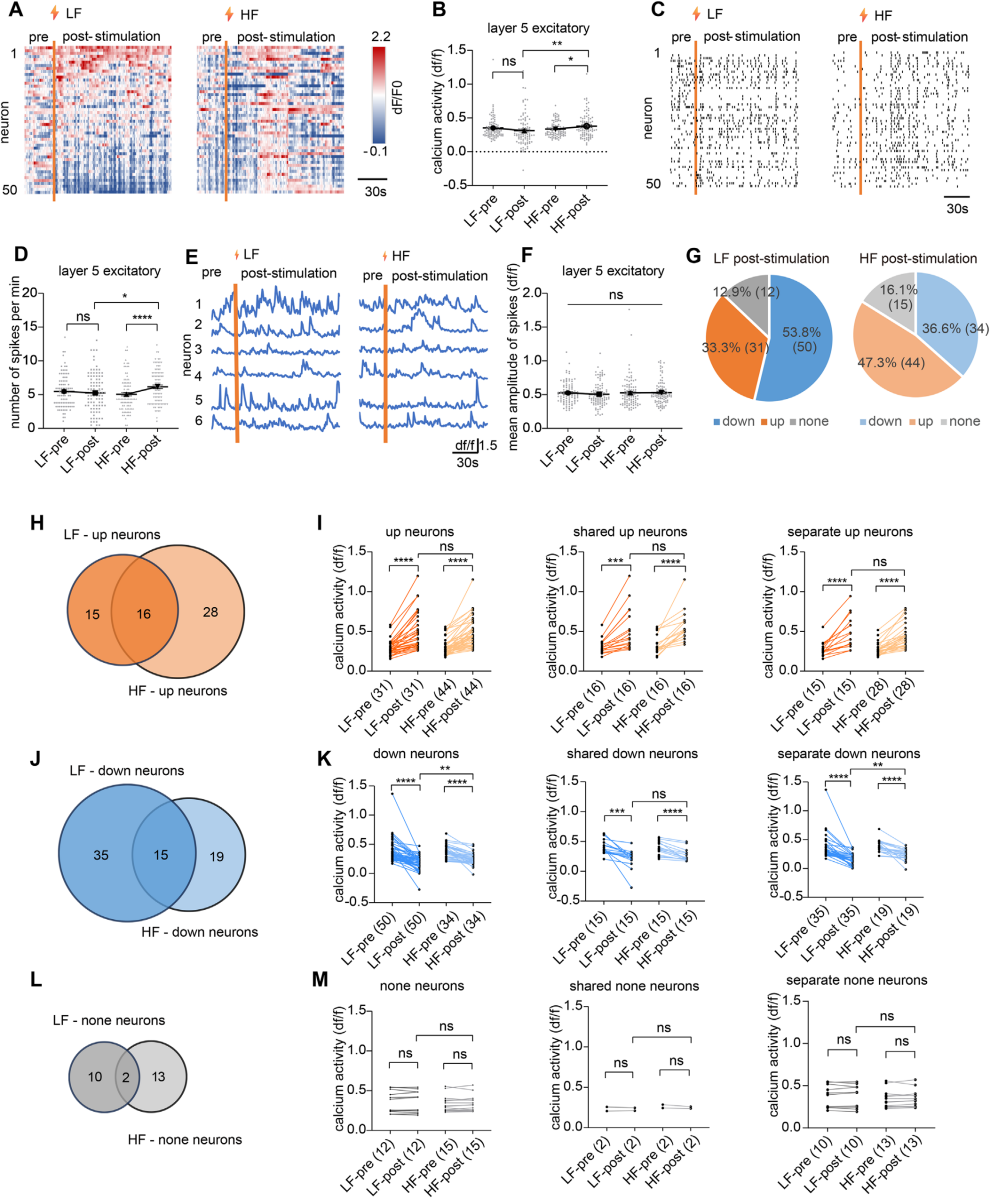
HF enhanced excitatory neuronal activity in layer 5 after stimulation cessation. (A) Heatmap of Ca^2+^ activity from 50 neurons before and after stimulation. (B) Ca^2+^ activity of layer 5 excitatory neurons before and after stimulation (*n* = 93 neurons from one mouse). (C) Ca^2+^ activity spikes from 50 excitatory neurons in layer 5 before and after stimulation. (D) Numbers of spikes per minute of layer 5 excitatory neurons before and after stimulation (*n* = 93 neurons from one mouse). (E) Example of Ca^2+^ traces from six neurons before and after stimulation. (F) Mean amplitude of spikes of layer 5 excitatory neurons before and after stimulation (*n* = 93 neurons from one mouse). (G) Pie charts of the number and proportion of neurons whose Ca^2+^ activity increased, remained unchanged, and decreased after stimulation at LF or HF. (H) Venn diagram of layer 2/3 excitatory neurons with increased Ca^2+^ activity during LF or HF stimulation. (I) ΔCa^2+^ activity of Up neurons, shared Up neurons, and separate Up neurons after LF or HF stimulation. (J) Venn diagram of layer 2/3 excitatory neurons with decreased Ca^2+^ activity during LF or HF stimulation. (K) ΔCa^2+^ activity of Down neurons, shared Down neurons, and separate Down neurons after LF or HF stimulation. (L) Venn diagram of layer 5 excitatory neurons staying unchanged after LF or HF stimulation. (M) ΔCa^2+^ activity of none neurons, shared none neurons, and separate none neurons after LF or HF stimulation. *p < 0.05, **p < 0.01, ***p < 0.001, ****p < 0.0001. Data are represented as mean ± SEM.

We then examined the effects of electric stimulations on layer 5 pyramidal neurons after the termination of stimulation (stimulation off vs. baseline) to evaluate any lasting effect of a short electrical stimulation. Compared to no rise over baseline activity during stimulation, HF induced delayed increase in overall activity (*p* = 0.048; Figure 4A,B) after stimulation cessation, with significant increase in spike frequency (*p* < 0.001; Figure 4C,D) but not peak amplitude (*p* = 0.186; Figure 4E,F). Compared to mild inhibition over baseline during stimulation, LF induced comparable activity with baseline after stimulation cessation (*p* = 0.070; Figure 4A,B). Response patterns were also analyzed, with Up neurons being the major population under HF stimulation and Down neurons being the major population under LF stimulation (Figure 4G). Shared Up and separate Up neurons displayed similar activity increase between HF and LF (Figure 4H,I). Shared Down neurons displayed similar activity decrease between HF and LF (*p* = 0.639; Figure 4J,K), but separate Down neurons were more inhibited under LF stimulations (*p* = 0.006; Figure 4J,K), suggesting that HF stimulation is more potent in elevating excitatory neuronal activity, and LF stimulation is strongly effective at suppressing excitatory neuronal activity. Remaining neurons displayed no significant changes under either LF or HF stimulation (Figure 4L,M). In summary, for layer 5 pyramidal neurons, compared with during stimulation, HF and LF continue to increase overall Ca^2+^ activity after stimulation cessation. Compared with baseline, HF induced a net increase in layer 5 excitatory neurons, while LF resulted in a relatively similar activity level in these neurons. Given that layer 2/3 neurons showed net increase in activity under both HF and LF during this stage, these results suggest that layer 5 pyramidal neurons have greater resistance to excitation compared to superficial layers.

### 2.5 HF and LF Enhanced Inhibitory Neuronal Activity During Stimulation

**FIGURE 5:**
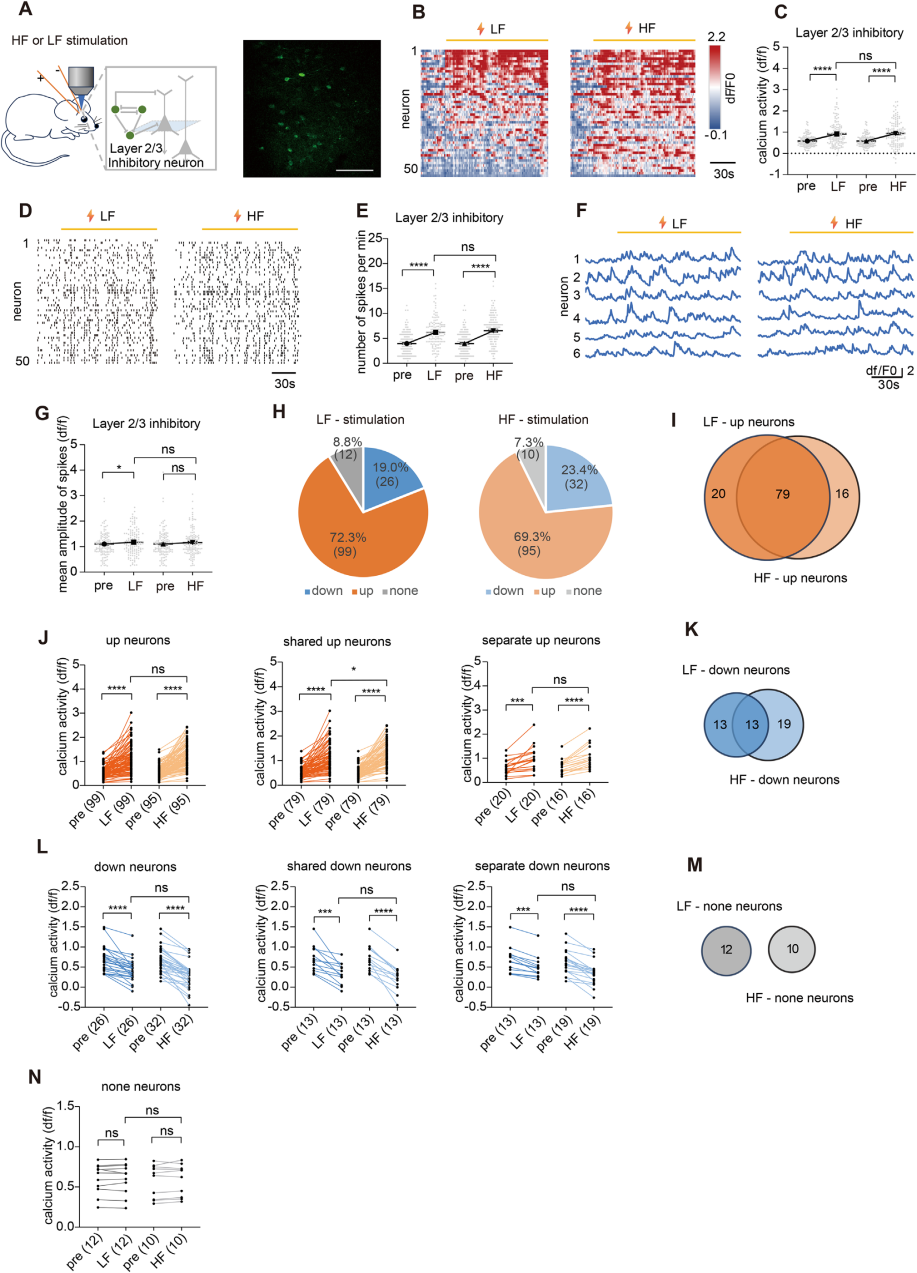
HF and LF enhanced inhibitory neuronal activity during stimulation. (A) Scheme of the experimental design and the example of two‐photon image of layer 2/3 inhibitory neurons. Scale bar, 100 µm. (B) Example heatmap of Ca^2+^ activity from 50 neurons before and during pulse stimulation. (C) Ca^2+^ activity of layer 2/3 inhibitory neurons before and during stimulation (*n* = 137 neurons from two mice). (D) Ca^2+^ activity spikes from 50 inhibitory neurons in layer 2/3 before and during stimulation. (E) Numbers of spikes per minute of layer 2/3 inhibitory neurons before and during stimulation (*n* = 137 neurons from two mice). (F) Example of Ca^2+^ traces from six neurons before and during stimulation. (G) Mean amplitude of spikes before and during stimulation (*n* = 137 neurons from two mice). (H) Pie charts of the number and proportion of neurons whose Ca^2+^ activity increased, remained unchanged, and decreased during LF or HF stimulation. (I) Venn diagram of layer 2/3 inhibitory neurons with increased Ca^2+^ activity during LF or HF stimulation. (J) Ca^2+^ activity of Up neurons, shared Up neurons, and separate Up neurons before and during LF or HF stimulation. (K) Venn diagram of layer 2/3 inhibitory neurons with decreased Ca^2+^ activity during LF or HF stimulation. (L) Ca^2+^ activity of Down neurons, shared Down neurons, and separate Down neurons before and during LF or HF stimulation. (M) Venn diagram of layer 2/3 inhibitory neurons with no change during LF or HF stimulation. (N) Ca^2+^ activity of none neurons before and during LF or HF stimulation. *p < 0.05, **p < 0.01, ***p < 0.001, ****p < 0.0001. Data are represented as mean ± SEM.

During electrical stimulation, we first analyzed the effects of LF and HF stimulation on inhibitory neuronal Ca^2+^ activity in layer 2/3 of S1 cortex (stimulation on vs. baseline). Results showed that both LF and HF stimulation significantly increased the inhibitory neuronal Ca^2+^ activity (*p* < 0.001; Figure 5A–C) and spike frequency (*p* < 0.001; Figure 5D,E). LF stimulation was found to have an enhancement effect on spike amplitude (*p* = 0.020; Figure 5F,G). Both LF and HF stimulation increased Ca^2+^ activity in a similar number of neurons (Figure 5H), with the majority being the same neurons (79/99 for LF and 79/95 for HF; Figure 5I). The Up neurons showed a similar increase between LF and HF, with the separate Up neurons displaying similar excitation, but shared Up neurons were mildly more activated under HF stimulations (*p* = 0.032; Figure 5I,J). The Down neurons showed similar inhibition between LF and HF, with the shared Down and separate Down neurons displaying a similar decrease (Figure 5K,L). The remaining neurons showed no significant changes under either LF or HF stimulation (Figure 5 M,N). In summary, both LF and HF enhanced Ca^2^^+^ activity in inhibitory neurons, with a large overlap in the responsive neuronal populations. Notably, the proportions of shared and separately modulated neurons were consistent between the two stimulation paradigms, suggesting that inhibitory neurons respond similarly to both LF and HF stimulation. Furthermore, the number of activated neurons far exceeded that of inhibited neurons, indicating that electrical stimulation predominantly enhances inhibitory neuronal activity. Overall, these findings suggest that inhibitory neuronal responses are largely frequency insensitive, with both LF and HF stimulation exerting similar effects—primarily increasing neuronal activation rather than inhibiting.

### 2.6 LF and HF Exert Lasting Effects on Neuronal Activity Coherence

**FIGURE 6:**
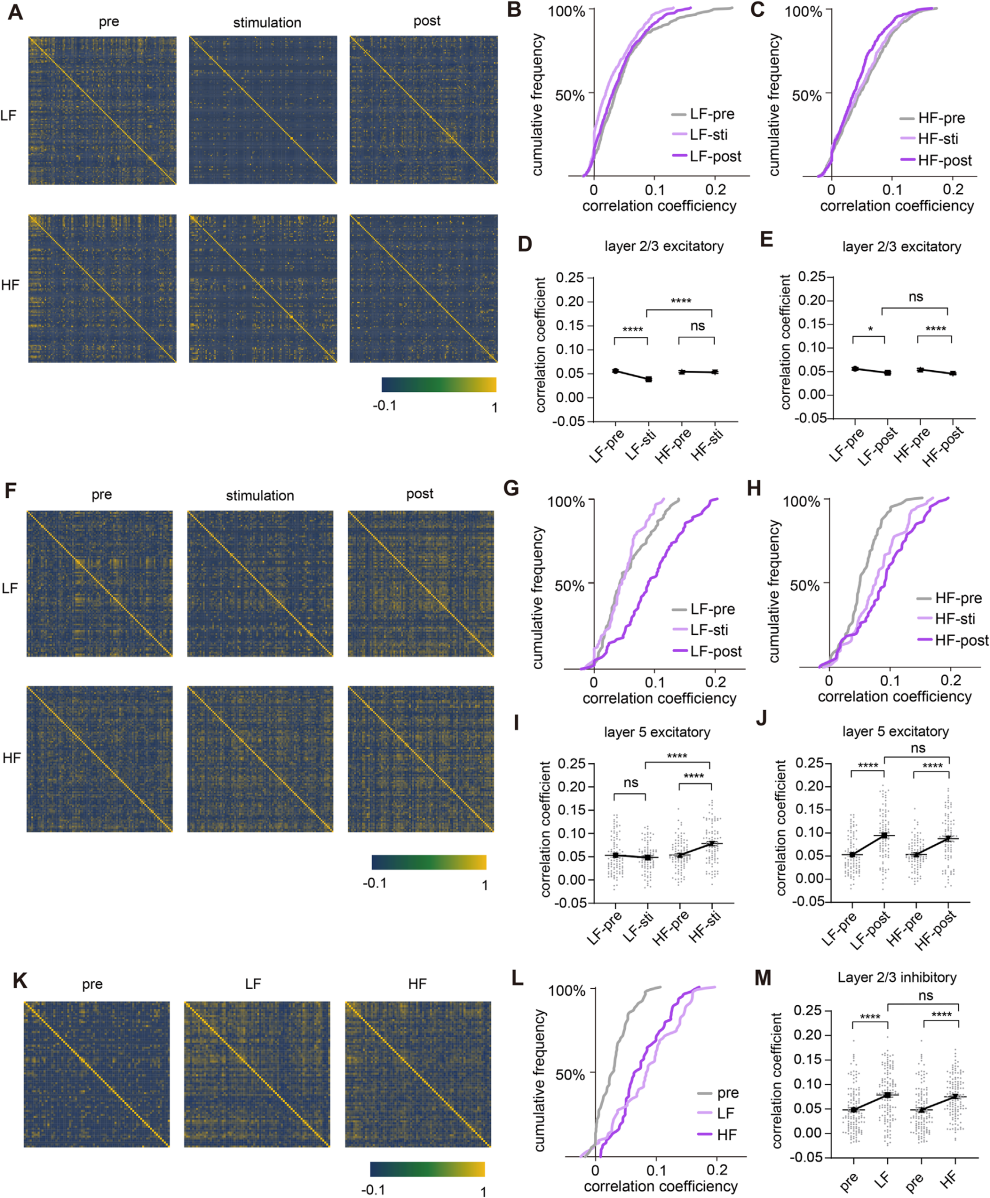
LF and HF exert lasting effects on neuronal activity coherence. (A) Representative correlation coefficient matrix of Ca^2+^ activity from layer 2/3 excitatory neuronal pairs before, during, and after LF or HF stimulation. (B) Cumulative frequency distribution of correlation coefficients of layer 2/3 excitatory neuronal pairs before, during, and post LF stimulation. (C) Cumulative frequency distribution of correlation coefficients of layer 2/3 excitatory neuronal pairs before, during, and post HF stimulation. (D) Correlation coefficients of layer 2/3 excitatory neurons before and during stimulation. (E) Correlation coefficients of layer 2/3 excitatory neurons before and post stimulation. (F) Representative correlation coefficient matrix of Ca^2+^ activity from layer 5 excitatory neuronal pairs before, during, and after LF or HF stimulation. (G) Cumulative frequency distribution of correlation coefficients of layer 5 excitatory neuronal pairs before, during, and post LF stimulation. (H) Cumulative frequency distribution of correlation coefficients of layer 5 excitatory neuronal pairs before, during, and post HF stimulation. (I) Correlation coefficients of layer 5 excitatory neurons before and during stimulation. (J) Correlation coefficients of layer 5 excitatory neurons before and post stimulation. (K) Representative correlation coefficient matrix of Ca^2+^ activity from layer 2/3 inhibitory neuronal pairs before and during stimulation. (L) Cumulative frequency distribution of correlation coefficients of layer 2/3 inhibitory neuronal pairs before and during stimulation. (M) Correlation coefficients of layer 2/3 inhibitory neurons before and during stimulation. *p < 0.05, **p < 0.01, ***p < 0.001, ****p < 0.0001. Data are represented as mean ± SEM.

Finally, we analyzed the activity correlation coefficients of different populations of neurons based on spike synchrony before, during, and post stimulation. We first examined the synchrony of layer 2/3 excitatory neurons (Figure 6A–C). The correlation coefficients of excitatory neurons in layer 2/3 were decreased during and post LF stimulation (Figure 6D,E). However, correlation coefficients were unchanged during HF stimulation and decreased after stimulation was stopped, suggesting that the degree of neuronal synchronization was reduced only after HF stimulation cessation (Figure 6D,E). In summary, electrical stimulations tend to desynchronize layer 2/3 excitatory neuronal activity, with a stronger effect of LF. In layer 5 excitatory neurons (Figure 6F–H), correlation coefficients were increased during and after HF stimulation (Figure 6I,J), while unchanged during LF stimulation and increased after stimulation ceased, suggesting that the degree of neuronal synchronization was only increased after the cessation of LF stimulation (Figure 6I,J). In summary, LF and HF increased the degree of synchronization in layer 5 excitatory neurons, with a stronger HF effect. In layer 2/3 inhibitory neurons, both LF and HF caused a similar increase in correlation coefficients, suggesting a higher level of synchronization (Figure 6K–M). Overall, on the same population of neurons, the effects of LF and HF on activity coherence were isotropic and long lasting, with some aftereffects.

## 3 Discussion

In our study, we observed that the regulatory effect of both 60 Hz (LF) and 160 Hz (HF) electrical stimulation on the excitatory and inhibitory neurons of S1 cortex was to enhance their Ca^2+^ activities by regulating the number of Ca^2+^ spikes, regardless of whether its effect was activated or inhibited. This is consistent with previous work using fMRI to record blood oxygen level‐dependent (BOLD) responses and finding increased neural network activity in large non‐primate mammals stimulated by STN‐DBS [20], indicating an overall activation. Frequency‐dependent STN‐DBS‐induced strong positive BOLD responses at multiple sites in the STN and basal ganglia‐thalamocortical network have also been observed in a Parkinsonian rat model [21]. In the resting state, excitatory or inhibitory neurons exhibit their own intrinsic spontaneous activity rhythms, which are essential for maintaining normal neural function and information transmission. LF and HF enhanced neuronal activity mainly by altering the spike frequencies. Though we observed that layer 2/3 neuronal spike amplitude was affected by pulsed electrical stimulation, it did not persist after stimulation cessation, suggesting that it is the direct transient effect of the current that does not have a sustained effect on the intrinsic activity of the neuron, and that it is the change in the neuronal spike frequency being the main effect of pulsed electrical stimulation.

Our results show that excitatory neurons of layer 2/3 are more susceptible to increased Ca^2+^ activity by pulsed electrical stimulation than excitatory neurons of layer 5. Previously, it has been found that most of the neurons in layer 2/3 maintain very low basal spontaneous firing rates, and only a very small number of them are high firing rate neurons; the population response exhibits a lognormal distribution [22], which may contribute to the highly selective encoding and response of S1 to different stimuli. One study simultaneously recorded the laminar structure of spontaneous group activity in rat auditory cortex and found that the spontaneous firing of putative pyramidal neurons in layer 2/3 was much sparser than in layer 5 [23]. In a sensorimotor decision‐making task in awake mice, the proportion of excitatory neurons in layer 2/3 that responded to stimuli with evoked discharges was also much lower than in layer 5 [24]. This is consistent with our observation that excitatory neurons in layer 2/3 have lower spontaneous discharges in physiological states, and explains why this group of neurons may be more susceptible when subjected to pulsed electrical stimulation. Excitatory neurons in L5 typically have higher firing rates compared to L2/3, which may make them less responsive to additional pulsed electrical stimulation.

Moreover, electrical stimulation activates axons within a certain range and modulates the activity of downstream brain regions by releasing neurotransmitters [25, 26]. The impact of electrical stimulation appears to depend on the relative distribution of excitatory and inhibitory inputs converging on the target neurons [27]. Thalamic structures, which predominantly receive excitatory inputs, exhibit excitatory neuronal responses following LF stimulation, whereas basal ganglia structures, primarily receiving inhibitory inputs, display inhibitory responses with stimulation [28, 29]. In our study, excitatory and inhibitory neurons in layer 2/3 of the S1 cortex received predominantly excitatory inputs [30, 31], which supports their elevated activity during electrical stimulation. Besides, in layer 2/3, excitatory neurons exhibited increased Ca^2+^ activity during HF stimulation, while Ca^2+^ activity began to rise only after the cessation of LF stimulation. This may be attributed to the higher charge accumulation within the HF stimulation at the same pulse width (90 µs), leading to a more pronounced effect on neuronal activity compared to LF stimulation.

We found that electrical stimulation induced distinct changes in neuronal synchrony across layers and cell types. Synchrony was elevated in layer 5 excitatory neurons and layer 2/3 inhibitory neurons, both of which exhibited relatively high spontaneous spike frequencies pre‐stimulation. In contrast, synchrony was weakened in layer 2/3 excitatory neurons, which displayed lower spontaneous firing pre‐stimulation. These data suggest that the effect of stimulation on synchrony may depend more on the intrinsic firing properties of cells rather than location or broad cell types.

These frequency‐ and layer‐specific alterations in synchrony may also provide a mechanistic link to the clinical effects of electrical stimulation. In Parkinson's disease (PD), HF‐DBS (130 Hz) of the STN alleviated motor symptoms—arising from chair, posture, and postural instability [32], while LF‐DBS (60 Hz) improves swallowing function and freezing of gait [33]. Although our stimulation was applied to the S1, the principle that frequency could systematically reshape synchrony in a layer‐specific manner may be broadly relevant. Pathological beta‐band synchronization is a hallmark of PD circuit dysfunction [19]. In our experiments, HF stimulation enhanced synchrony in layer 5 excitatory neurons—a major cortical output layer. Therefore, HF‐DBS may act in part by imposing a regulated synchronous rhythm that suppresses pathological network dynamics. Conversely, the pronounced desynchronization of L2/3 excitatory neurons by LF stimulation could relate to the cognitive or perceptual side effects occasionally seen with non‐therapeutic low‐frequency stimulation [12].

Our findings may also shed light on the potential mechanisms of stimulation in treating chronic pain. In pain states, synchronized activity in primary sensory neurons can initiate cortical synchrony in S1 [17], and pain itself induces stable, hyperactive microcircuits in this region [18]. Previous studies have shown that pain conditions increase synchronization among S1 layer 2/3 excitatory neurons, and that artificially enhancing S1 synchrony can lower pain thresholds [18]. In this context, our finding that pulsed electrical stimulation robustly reduces synchrony in L2/3 excitatory neurons—while concurrently enhancing it in local inhibitory networks—suggests a potential therapeutic mechanism. By targeted disruption of pain‐associated hypersynchrony and potentially rebalancing local E‐I dynamics, electrical stimulation may help normalize pathological circuit activity.

In addition to neurons, glial cells in S1 may also participate in the modulation of pulse electrical stimulation [34]. Astrocytes play an active role in neuronal communication, and the astrocytic network can regulate neuronal activity [35]. Astrocytes exhibit rapid increases in Ca^2+^ levels in response to electrical stimulation, indicating their responsiveness to such stimulation [36]. Activated astrocytes may influence neuronal activity through the secretion of adenosine and glutamate [37, 38]. Interactions between glial cells and nerve cells during electrical stimulation remain to be elucidated.

While our study provides insights into the frequency‐dependent and layer‐specific effects of pulsed electrical stimulation, several methodological limitations should be considered. First, it is important to note that the kinetics of GCaMP6s can lead to signal saturation and a loss of linear correspondence with action potentials at very high firing rates [39]. Therefore, our neuronal Ca^2^^+^ signals should be interpreted as an integrated measure of neuronal activation rather than a linear readout of instantaneous firing rate. Future studies employing simultaneous electrophysiology will be valuable to precisely delineate the spike–Ca^2^^+^ relationship under these stimulation paradigms. Second, imaging of the L2/3 and L5 neurons was not performed simultaneously. Therefore, we did not have precise intracortical microloop evidence to compare the effects of HF versus LF electrical stimulations. Third, the recorded neurons in this study were recorded at the depth of 500–800 µm in the S1HL region, mainly comprising the superficial L5a, and as our imaging was performed on a single optical plane near the stimulation electrode, we did not capture the full three‐dimensional extent of the activated network and potential long‐range effects. It should be noted that this study did not track the post‐stimulation activity of inhibitory neurons. Determining whether inhibitory circuits exhibit sustained activation, rapid suppression, or cell‐type‐specific rebound dynamics following stimulation is critical, which merits future studies for understanding whether E/I balance is affected and contributes to the lasting effects of neuromodulation.

## 4 Conclusion

Our results found frequency‐specific and layer‐specific modulation effects of electrical stimulation, providing insights into the cellular mechanisms underlying electrical stimulation and its potential therapeutic applications for neuropsychiatric disorders.

## 5 Materials and Methods

### 5.1 Animals

Male C57/BL6 mice with initial weights of 20–22 g were provided by Experimental Animal Center of Peking University. Animals (six mice per cage) were housed in barrier condition with inversed 12‐h light–dark cycle where temperature and humidity were well controlled (22 ± 1°C, 50 ± 5%). Food and water were available ad libitum, and padding was renewed regularly. All experimental procedures were approved by the Animal Use Committee of Peking University Science Center, conformed to ethics of laboratory animal, and followed the instructions of National Institutes of Health Guide for the Care and Use of Laboratory Animals.

### 5.2 Virus

The pAAV‐CaMKIIα‐GCaMP6s‐WPRE (titer 1.3E+12 v.g./mL, OBiO Technology (Shanghai) Corp., Ltd.) and pAAV‐GAD67‐GCaMP6s‐3xFLAG (titer 1.7E+12 v.g./mL, OBiO Technology (Shanghai) Corp., Ltd.) were employed for transfecting excitatory and inhibitory neurons separately.

### 5.3 Viral Injection

Viral injections were conducted in 8‐ to 10‐week‐old mice. Mice were anesthetized via intraperitoneal injection of 15 mL/kg of 4% tribromoethanol. Erythromycin ophthalmic ointment (Guangzhou Baiyunshan Pharmaceutical Co., Ltd., Guangzhou, China) was applied to protect the eyes, and fur from the head was shaved. Once the mice were secured in a stereotaxic device, the scalp was sterilized with iodophor. A 1‐cm incision was made in the scalp, and connective tissue was carefully removed to expose the skull. After thorough drying, a handheld drill was used to create small holes in the skull above the S1 (AP = −0.5 mm, ML = ±1.5 mm) on both sides, ensuring that the dura mater was not damaged. Subsequently, a Hamilton syringe with a glass microelectrode attached was used for viral injections through the drilled holes, targeting depths of 200 µm and 500 µm from the dura mater, with a total injection volume of 200 nL on each side. The needle was held in position for approximately 10 min post‐injection before being slowly retracted. The scalp was sutured, and erythromycin ophthalmic ointment was applied to the wound to prevent infection. Mice were allowed a recovery period of 4 weeks before imaging.

### 5.4 Cranial Window Preparation

Mice were anesthetized with 6 mL/kg of 4% tribromoethanol. After shaving and sterilizing the head, the skin was incised to expose the skull, which was meticulously cleaned and dried by removing any adhering membranes and fatty tissue. Dental cement was applied along the two long edges of a custom‐made rectangular stainless steel frame, centered over the S1, and affixed to the skull, leaving an 8‐mm gap in the middle of the cement edges. Once the dental cement was fully cured, a circular trajectory approximately 2.5 mm in diameter was drilled into the skull surface directly above S1 using a handheld drill. The notch was gradually deepened until the dura mater was exposed, carefully removing the circular bone fragments with forceps. Throughout this process, a 10‐mL syringe filled with artificial cerebrospinal fluid (ACSF) was used to flush the area, preventing any potential active bleeding. The exposed brain tissue was covered with a droplet of ACSF to maintain cellular viability. A 3‐mm diameter glass coverslip was placed over the craniotomy to ensure complete coverage of the skull. After removing excess ACSF with absorbent paper, a ring of 502 glue was applied at the junction of the coverslip and skull, and allowed to dry for 5 min to prevent ACSF leakage and ensure there was no active bleeding on the brain surface.

### 5.5 Electric Implant and Stimulation in the S1 Cortex

PINS‐T901 test stimulator was used. Voltage‐controlled, cathodic‐leading, charge‐balanced pulsed currents of 1 V, 100 µA, 90 µs pulse width were delivered via the test stimulator with the frequency of 60 Hz (LF) or 160 Hz (HF). To facilitate the current passage through the S1 cortex, a small hole was drilled approximately 2 mm lateral to the cranial window, and a corresponding hole was drilled symmetrically on the opposite side. Insulated metal wires, with only the tips exposed for conductivity, were angled through each of the holes to penetrate the S1 cortex. Dental cement was used to secure the wires to the skull surface. Finally, additional dental cement was applied to connect the previously affixed edges of the metal frame to form a stable closed loop. The PINS‐T901 test stimulator was connected to the metal wires on either side of the cranial window. The mice were used for imaging once the dental cement had fully dried and they were completely awake.

### 5.6 Two‐Photon Ca^2+^ Imaging

Two‐photon imaging was conducted using a TCS‐SP8 DIVE microscope (Leica, Germany) equipped with a tunable‐wavelength laser source (680‐1300 nm, MaiTai DeepZ). Excitation at 920 nm was applied according to the viral constructs utilized, with a water immersion objective (APO 25×/0.95). Mice were immobilized on an in vivo imaging platform, with the cranial window securely coupled to a 20× water immersion objective lens using an aqueous gel. Cells recorded at depths 150–300 µm were taken as layer 2/3, and cells at depths 500–800 µm were taken as layer 5. Each image was captured at a resolution of 512 × 512 pixels (738.1 × 738.1 µm), with an acquisition frequency of 0.653 s/frame (1.53 fps).

Awake mice were initially immobilized on the imaging platform for 15 min to acclimatize. A 2‐min baseline activity, followed by a 2‐min of LF electric stimulation and two more minutes of post‐stimulation, was recorded. After a 5 ‐ min rest period, a 2 ‐ min baseline, followed by 2 min of HF electrical stimulation and a 2 ‐ min post ‐ stimulation period, were recorded.

### 5.7 Data Analysis

The collected two‐photon images were analyzed using ImageJ. After motion correction of the image sequences using the Image Stabilizer plugin, the experimenter manually outlined the neuronal soma regions in the sequences of LF and HF stimulation, enabling the matching of Ca^2+^ activity data from the same neurons. After extracting the fluorescence signals from individual cells, data processing was performed using MATLAB R2022b using Github code for GCAMP deconvolution deposited by Pengcheng Zhou [40]. The average fluorescence intensity of the lowest 10% during baseline imaging was taken as F0, and the ΔF/F0 for each frame was calculated. The average ΔF/F0 values for baseline, stimulation, and post‐stimulation were calculated, respectively. Neurons with the increase in ΔF/F0 of ≥ 10% over baseline during electric stimulation were classified as “up,” while those with the decrease of ≥ 10% were classified as “down.” Neurons showing no significant change between these two thresholds were classified as “none.” After stimulus cessation, neurons with Ca^2+^ activity increased by ≥ 10% over baseline were classified as “activated,” neurons with a decrease of ≥ 10% or more were classified as “inhibited,” and neurons with no significant change were classified as “unchanged.” The noise floor of each cell was defined as the average of lowest 10% of the ΔF/F0 over the 2‐min baseline. A spike was defined if the peak ΔF/F0 is greater than three times the standard deviation of the noise floor of that neuron. The spike frequency and amplitude were separately calculated before, during, and after stimulation. The Ca^2+^ activity correlation coefficients between neurons were analyzed based on the number and time distribution of spikes before, during, and after stimulation, and the average value was used as a parameter to reflect synchrony. Statistical analysis and graphing were performed using GraphPad Prism 8 software.

## Author Contributions

Linlin Sun conceived the project. Linlin Sun, Zhiyan Wang, and Xiyao Gu supervised all experiments. Xinzhi Ye and Junfeng Wang performed the experiments and wrote the manuscript. Xinzhi Ye, Junfeng Wang, Jiao Liu, Zepeng Liu, Yuxin Huang, Wei li, and Jiaxin Wang analyzed the data. All authors discussed the results and contributed to the final manuscript. Xiyao Gu, Zhiyan Wang, and Linlin Sun revised the manuscript. All authors have read and approved the final manuscript.

## Conflicts of Interest

The authors declare no conflicts of interest.

## Funding

This work was supported by the National Natural Science Foundation of China grants 82101231 (to L.S.), Beijing Natural Science Foundation of China grant 7252082 (to L.S.), Peking University Talent Fund 68263Y1230 (to L.S.), Natural Science Foundation of Shanghai 25ZR1401225 (to X.G.), and Talent Fund of Shandong First Medical University 200025 (to Z.W.).

## Ethics Statement

All animal experiments were approved by members of the Laboratory Animal Ethics Sub‐Committee of the Medical Ethics Committee of Peking University, approval number LA2022376.

## Data Availability

All data that support the findings of this study are available from the corresponding author upon reasonable request.
